# A Case of Postoperative Wernicke Encephalopathy Mimicking Cerebellar Infarction Complicated by Postoperative Nausea and Vomiting: A Critical Diagnostic Pitfall for Thoracic Surgeons

**DOI:** 10.70352/scrj.cr.25-0438

**Published:** 2025-11-18

**Authors:** Tomonari Oki, Shuhei Iizuka, Takashi Saito, Toru Nakamura

**Affiliations:** 1 Department of General Thoracic Surgery, Seirei Hamamatsu General Hospital, Hamamatsu, Shizuoka, Japan; 2 Department of Neurology, Seirei Hamamatsu General Hospital, Hamamatsu, Shizuoka, Japan

**Keywords:** Wernicke encephalopathy, pulmonary lobectomy, postoperative nausea and vomiting, cerebral infarction, malnutrition

## Abstract

**INTRODUCTION:**

Wernicke encephalopathy (WE) is a potentially life-threatening neurological disorder caused by a thiamine deficiency, most commonly associated with alcoholism or malnutrition. Although its occurrence after gastrointestinal surgery has been increasingly recognized, WE following thoracic surgery remains extremely rare and is often underrecognized by thoracic surgeons. Given that neurological symptoms of WE can mimic those of cerebral infarction, a timely diagnosis is challenging, especially when complicated by postoperative nausea and vomiting (PONV).

**CASE PRESENTATION:**

We report a rare case of WE following a pulmonary lobectomy in a severely malnourished 75-year-old woman with a history of recurrent thyroid cancer. A 1.6-cm pulmonary nodule was incidentally detected during routine follow-up, and a diagnosis of a primary lung adenocarcinoma was established. The patient underwent a right upper lobectomy and mediastinal lymph node dissection without intraoperative complications. Persistent PONV developed immediately postoperatively, necessitating peripheral parenteral nutrition without vitamin supplementation. On POD 3, the patient developed ataxia, dysmetria, and saccadic eye movements. Although a cerebral infarction was initially suspected, brain magnetic resonance imaging revealed hyperintense signals in the periaqueductal region, suggestive of WE. Intravenous thiamine replacement with fursultiamine led to the gradual resolution of the neurological symptoms and nausea. The patient resumed oral intake on POD 7 and was discharged on day 10 without any neurological sequelae.

**CONCLUSIONS:**

This case highlighted the importance of considering WE as a differential diagnosis in malnourished patients presenting with neurological symptoms and PONV after a pulmonary resection, even in the absence of alcohol use. Early recognition and empiric thiamine supplementation are crucial to prevent irreversible neurological damage. Thoracic surgeons should maintain a high index of suspicion for WE, particularly in at-risk patients, to avoid a delayed diagnosis and improve clinical outcomes.

## Abbreviations

BMI
: body mass index
DWI
: diffusion-weighted imaging
ERAS
: enhanced recovery after surgery
FLAIR
: fluid-attenuated inversion recovery
GNRI
: geriatric nutritional risk index
PAX8
: paired box gene 8
PNI
: prognostic nutritional index
PONV
: postoperative nausea and vomiting
TTF-1
: thyroid transcription factor-1
WE
: Wernicke encephalopathy

## INTRODUCTION

Wernicke encephalopathy (WE) is a serious neurological disorder caused by a thiamine (vitamin B1) deficiency, which can progress to Korsakoff syndrome, leading to irreversible neurological damage or death if not promptly treated. Although WE is classically associated with chronic alcoholism and malnutrition, recent reports have highlighted its occurrence following gastrointestinal surgery due to postoperative malnutrition or prolonged nausea and vomiting. In contrast, WE is exceedingly rare following thoracic surgeries and remains unrecognized by thoracic surgeons. This rarity is likely due to the widespread adoption of enhanced recovery after surgery (ERAS) protocols, which promote early postoperative oral intake and mitigate the risk of nutritional deficiencies.

While thoracic surgeons are familiar with cerebral infarction caused by thrombus formation at the pulmonary vein stump as a common postoperative complication, its neurological symptoms can mimic those of WE, making a timely diagnosis challenging. Moreover, postoperative nausea and vomiting (PONV), another common complication, can further complicate the diagnosis due to overlapping clinical findings. Herein, we present a rare case of WE following a pulmonary lobectomy, highlighting the diagnostic pitfalls as well as the importance of early preventive and therapeutic interventions.

## CASE PRESENTATION

**Fig. 1:**
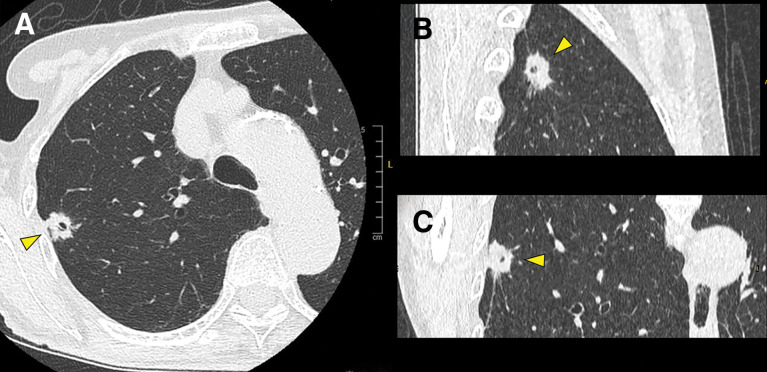
Chest CT. A 1.6-cm subpleural nodule with irregular margins, spiculation, and an internal cavity (yellow arrowheads) is visible in the right upper lobe. (**A**) Axial view, (**B**) sagittal view, and (**C**) coronal view.

A 75-year-old woman, with no history of smoking or alcohol consumption, was found to have a 1.6-cm solid nodule in the right upper lobe of the lung during routine follow-up CT after surgical treatment for a papillary thyroid carcinoma (pT4aN1bM0 stage III) (**Fig. 1**). Seventeen years earlier, she had undergone a subtotal thyroidectomy and cervical lymph node dissection for thyroid carcinoma. Five years after the initial surgery, recurrence in the right paratracheal lymph nodes required a resection of the thyroid remnant and lymph node dissection. Six years later, recurrent metastases to the right deep cervical lymph nodes necessitated a total of 5 additional lymph node dissections over 4 years. Following these interventions, local disease control had been achieved without any further recurrence for the past 2 years. She had been receiving thyroid hormone replacement therapy following total thyroidectomy, and her thyroid hormone levels were well controlled within the normal range.

The patient was severely underweight, with a height of 160 cm, weight of 39 kg, and body mass index (BMI) of 15.2. Although her serum albumin level remained within the normal range at 4.1 g/dL, her Prognostic Nutritional Index (PNI) was 42.2, and her Geriatric Nutritional Risk Index (GNRI) was 89.3, indicating a preoperative nutritional risk.^1,2)^

Bronchoscopic biopsy of the pulmonary nodule revealed an adenocarcinoma. Immunohistochemical staining demonstrated positivity for thyroid transcription factor-1 (TTF-1) and negativity for paired box gene 8 (PAX8) and thyroglobulin, consistent with a diagnosis of a primary lung adenocarcinoma (T1bN0M0 stage IA2). Preoperative brain MRI revealed no pre-existing cerebral abnormalities.

The patient underwent a right upper lobectomy and mediastinal lymph node dissection under general anesthesia combined with epidural anesthesia containing opioids. The final pathological diagnosis was a papillary adenocarcinoma with an invasive component measuring 1.6 cm and visceral pleural invasion, classified as T2aN0M0 stage IB.

**Fig. 2:**
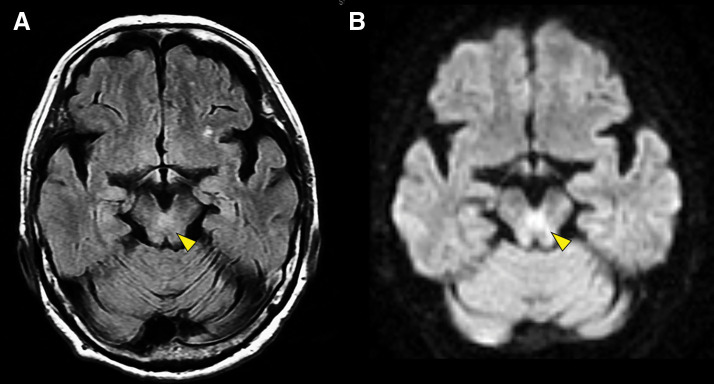
Brain MRI. Hyperintensity (yellow arrowheads) is demonstrated around the periaqueductal region of the midbrain on (**A**) fluid-attenuated inversion recovery and (**B**) diffusion-weighted imaging.

PONV developed immediately after surgery. Despite intravenous fluid supplementation, antiemetic administration, and discontinuation of epidural opioids, the symptoms persisted. Consequently, her oral intake was significantly limited, and peripheral parenteral nutrition without vitamin supplementation became necessary. On POD 3, the patient developed ataxia, dysmetria, and saccadic eye movements. No alteration in mental status was observed. Given the suspicion of a cerebral infarction, an urgent MRI was performed. Although no evidence of an acute cerebral infarction was detected, hyperintense signals were observed in the periaqueductal region of the midbrain on fluid-attenuated inversion recovery (FLAIR) and diffusion-weighted imaging (DWI) (**Fig. 2**).

Considering the characteristic MRI findings and clinical features, including malnutrition, ocular motor dysfunction, and cerebellar ataxia, a diagnosis of WE was established. At the time of diagnosis, serum thiamine levels were obtained; although results were not immediately available, they were later found to be low at 33 ng/mL (reference range: 24–66 ng/mL). Thiamine replacement therapy was initiated with intravenous fursultiamine, a thiamine derivative, at a dose of 200 mg/day for 5 days, which led to a gradual improvement in both the nausea and neurological manifestations. By POD 7, the patient was able to resume oral intake, and her neurological deficits had completely resolved. The patient was discharged on POD 10, and oral fursultiamine therapy (100 mg/day) was continued for 3 months. Although it took approximately 2 months for her appetite to resolve, she fully recovered and returned to her daily activities within 3 months, with no residual neurological abnormalities. One year postoperatively, no neurological sequelae were observed, and there was no progression to Korsakoff syndrome.

## DISCUSSION

WE is a potentially life-threatening neurological disorder caused by a thiamine (vitamin B1) deficiency. It is classically characterized by a triad of an altered mental status, ophthalmoplegia, and ataxia.^3,4)^ The estimated prevalence of WE ranges from 0.4% to 2.8%.^3)^ Although chronic alcoholism has traditionally been regarded as the most common cause, it accounts for only approximately half of all cases.^3)^ In contrast, non-alcoholic etiologies such as malnutrition secondary to prolonged vomiting, hyperemesis gravidarum, parenteral nutrition, gastrointestinal surgery, starvation, and endocrine disorders have been increasingly recognized.^3)^

Thiamine deficiency impairs both the Krebs cycle and the pentose phosphate pathway, resulting in cytotoxic and vasogenic cerebral edema.^5,6)^ MRI findings typically include T2-weighted and FLAIR hyperintensities in characteristic regions such as the paraventricular thalamus, hypothalamus, mammillary bodies, periaqueductal gray matter, and floor of the 4th ventricle.^7)^ Although MRI demonstrates approximately 90% specificity for WE, its sensitivity is limited to around 50%, and normal imaging findings do not exclude the diagnosis.^7)^ In clinical practice, CT is often performed for emergency head imaging. The CT findings of WE include low-density areas in the thalamus, midbrain, and pons; however, its sensitivity is only about 13%, making it less useful for diagnosis than MRI.^8)^ In addition, WE cannot be excluded even if serum thiamine levels are within the normal range, and due to the time-consuming nature of this test, it is not considered suitable for timely diagnosis. No single test provides definitive diagnostic confirmation.^9)^ Moreover, only 10% of patients present with the full triad of classical symptoms.^4)^ Therefore, clinicians must maintain a high index of suspicion for WE, particularly in malnourished patients presenting with relevant clinical features. Empiric thiamine supplementation should be promptly initiated once other potential etiologies, such as a cerebral infarction, have been reasonably excluded. Early intervention can lead to rapid symptom resolution, whereas untreated WE carries a mortality rate of approximately 20% and results in irreversible neurological sequelae, including Korsakoff syndrome, in up to 80% of survivors.^4,7)^

Severe malnutrition following a pulmonary resection is uncommon, largely due to the widespread implementation of ERAS protocols that facilitate early postoperative oral intake and reduce the risk of nutritional deficiencies.^10)^ Consequently, WE following a pulmonary resection is exceedingly rare and may often be underrecognized by thoracic surgeons.

When a patient develops nausea, ophthalmoplegia, and ataxia following a pulmonary lobectomy, thoracic surgeons must first consider the possibility of a cerebral infarction.^11)^ The incidence of cerebral infarction following a lobectomy ranges from 0.27% to 0.8%,^12–14)^ with cerebellar infarctions accounting for approximately 0.2%.^14)^ Notably, a left upper lobectomy carries a higher risk of postoperative cerebral infarction, likely due to thrombus formation at the pulmonary vein stump.^15,16)^

While thoracic surgeons are generally familiar with cerebral infarction as a potential postoperative complication, awareness of WE is limited due to its rarity. The similarity in the clinical presentation between these 2 conditions further complicates a timely diagnosis, often resulting in delayed treatment.

Moreover, PONV is commonly observed after a pulmonary resection, particularly in younger patients, females, non-smokers, individuals with a lower BMI, and those with a history of motion sickness,^17)^ and its incidence is approximately 30%, rising to nearly 40% in female patients.^18)^ PONV is common and may mask both cerebral infarction and the rare WE, hindering a timely diagnosis unless clinicians maintain a high index of suspicion.

The PNI and GNRI are useful indicators that reflect the preoperative nutritional status and predict postoperative complications and prognosis. Low PNI and GNRI values have been reported to be associated with an increased incidence of postoperative complications.^19,20)^ The PNI is calculated as 10 × serum albumin level (g/dL) + 0.005 × peripheral blood lymphocyte count (/mm^3^), and a value below 45 is considered high risk.^1)^ The GNRI is calculated as 14.89 × serum albumin level (g/dL) + 41.7 × (current body weight/ideal body weight). A score ≥98 is considered normal, 92 to <98 as low risk, 82 to <92 as moderate risk, and <82 as high risk.^20,21)^

In gastrointestinal surgery, preoperative prophylactic thiamine supplementation is considered for high-risk patients with pre-existing thiamine deficiency, malnutrition, or weight loss.^22,23)^ In thoracic surgery, severe preoperative starvation leading to thiamine deficiency is rare, and patients can typically resume oral intake soon after surgery; therefore, the necessity of routine preoperative prophylactic thiamine administration remains debatable. However, if high-risk patients experience delayed resumption of oral intake due to PONV, requiring prolonged parenteral nutrition, the administration of intravenous glucose without thiamine supplementation can induce WE.^23)^ Thus, we believe that thiamine supplementation before glucose administration is also beneficial in the context of thoracic surgery.

While total thyroidectomy does not directly cause thiamine deficiency, it has been reported to be a potential cause of malnutrition.^24)^ In this case, multiple thyroid surgeries, including a total thyroidectomy, may have contributed to chronic malnutrition, indirectly increasing the risk of WE. This was supported by her significantly low body weight and reduced PNI and GNRI, all of which indicated a poor nutritional status preoperatively.^1,2)^ While persistent PONV may have contributed to a reduced oral intake, it is more likely that the cumulative effects of repeated surgeries and chronic malnutrition predisposed her to a thiamine deficiency, ultimately leading to the development of WE. While a cerebellar infarction was initially suspected at symptom onset, WE was not even considered. Fortunately, characteristic MRI findings facilitated a timely diagnosis and treatment. However, given the limited sensitivity of MRI for WE, reliance solely on imaging can result in delayed diagnosis. This case underscores the importance of heightened awareness of WE among thoracic surgeons, particularly in malnourished patients without a history of alcohol consumption following a pulmonary resection.

## CONCLUSIONS

WE is an extremely rare but potentially fatal complication following pulmonary resection, particularly in malnourished patients, including those without a history of alcohol consumption. Because its clinical manifestations may overlap with more common postoperative complications such as cerebral infarction and PONV, early diagnosis can be challenging. This case highlights the need for thoracic surgeons to maintain a high index of suspicion for WE in at-risk patients and to initiate empiric thiamine replacement therapy promptly when clinical features are suggestive, even when imaging findings are inconclusive and serum thiamine levels are within the normal range.

## DECLARATIONS

### Availability of data and materials

Data sharing is not applicable to this article as no datasets were generated or analyzed during the current study.
